# High sugar intake exacerbates cardiac reperfusion injury in perinatal taurine depleted adult rats

**DOI:** 10.1186/1423-0127-17-S1-S22

**Published:** 2010-08-24

**Authors:** Supaporn Kulthinee, J Michael Wyss, Dusit Jirakulsomchok, Sanya Roysommuti

**Affiliations:** 1 Department of Physiology, Faculty of Medicine, Khon Kaen University, Khon Kaen 40002, Thailand; 2 Department of Cell Biology, School of Medicine, University of Alabama at Birmingham, Birmingham, AL 35294, USA

## Abstract

Perinatal taurine depletion and high sugar diets blunted baroreflex function and heightens sympathetic nerve activity in adult rats. Cardiac ischemia/reperfusion also produces these disorders and taurine treatment appears to improve these effects. This study tests the hypothesis that perinatal taurine exposure predisposes recovery from reperfusion injury in rats on either a basal or high sugar diet. Female Sprague-Dawley rats were fed normal rat chow with 3% beta-alanine (taurine depletion, TD), 3% taurine (taurine supplementation, TS) or water alone (control, C) from conception to weaning. Male offspring were fed normal rat chow and water containing 5% glucose (G) or water alone (W) throughout the experiment. At 7-8 weeks of age, all rats were anesthetized and their trachea clamped until cardiac arrest occurred and mean arterial pressure fell below 60 mm Hg. The clamp was immediately released and cardiopulmonary resuscitation was performed with cardiac function returning within 4 min. Twenty-four hours later, arterial pressure, heart rate, and baroreflex function were measured in conscious and one day later in anesthetized conditions. Basic blood chemistry and circulating markers of cardiac injury were also measured. Baroreflex sensitivity was depressed moderately in CG and TDW, and severely in TDG. TSW displayed increased baroreflex and high sugar intake returned it to CW. Sympathetic nerve activity increased and parasympathetic decreased in TDW but not TSW and these effects were exacerbated sharply in TDG and slightly in TSG. Arterial pressure and heart rate increased in all groups but to a lesser degree in TDG. Plasma aspartate aminotransferase increased in all groups except TSW, but the increase was nearly 3X greater in TDG vs. any other group. Creatine kinase-MB increased in all groups except TSG and was far greater in TD than other groups. Troponin-T and brain natriuretic peptide were greatly increased in TDG compared to all other groups. Thus, perinatal taurine depletion increases injury from cardiac ischemia/reperfusion, and in adult rats on a high sugar diet, these effects are greatly exacerbated.

## Introduction

Myocardial ischemia is the leading cause of death among people around the world [1,2]. Cardiac arrest also accounts for 20% of deaths following myocardial infarction, primarily in the first 1-7 days (75%) [3]. Myocardial ischemia has a close relation to the autonomic nervous system dysregulation; particularly heighten sympathetic nerve activity before symptoms and during recovery. In myocardial infarction patients, sympathetic nerve activity is elevated by a few days after ischemia (2–4 days) and remains high for 3-6 months [4]. Baroreceptor reflex control of heart rate is also blunted or resetting in these patients [5-8]. β-blockers are often prescribed for the treatment of patients with chronic heart failure [9] and myocardial infarction [10,11].

Taurine has been reported to improve cardiac function in many disorders. Plasma taurine increases in patients with acute myocardial ischemia and increases with the severity of the infarction [12]. Its source seems to be mainly from the myocardium. In this case, other cardiac peptides also increase, e.g., B-type natriuretic peptide [13]. The circulating taurine concentration in these patients increases for up to three days, paralleling the increased creatine kinase levels [12]. Taurine appears to be able to inhibit sympathetic nerve overactivity in these patients [14,15], and thus taurine may retard the rise in sympathetic nerve activity during ischemia and post cardiac reperfusion and protect the heart from adverse effects of sympathetic nerve hyperactivity and its consequences [16,17].

Taurine plays an important role in animals and humans at all age periods especially at the early life [18,19]. Prenatal taurine deficiency induces low birth weight in animals and the adult offspring are at high risk of cardiovascular diseases, diabetes mellitus, and renal damage [20]. Perinatal taurine supplementation, in contrast, attenuates hypertension in spontaneously hypertensive rats [21,22]. Previous experiments indicated that either taurine depletion or supplementation in early life altered renal function [23] and autonomic nervous control of arterial pressure [24] in adult rats. Their renal hemodynamics and excretory function are also modified by altered perinatal taurine exposure [23].

Perinatal taurine depletion depresses the autonomic function and blunts baroreflex function in adult male rats [24]. If these animals are treated by high sugar intake since weaning, the blunted baroreflex function will be exacerbated and sympathetic nerve activity is in turn heightened. Perinatal taurine supplementation does not possess this action. Blunted baroreflex function and sympathetic nerve overactivity are also observed in cardiac ischemia/reperfusion, and these changes appear to be reduced by taurine treatment [16,17]. Whether perinatal taurine over or under exposure predisposes the autonomic nervous system function and cardiac injury in this abnormality has not been established. The present study tests the hypothesis that perinatal taurine exposure increases the efficiency of autonomic nervous system control of arterial pressure at the early phase of recovery from cardiac arrest (2-5 days) in adults. Perinatal taurine supplementation or deficiency effects are compared to lifetime normal taurine diet control offspring. Their interactions with high sugar intake since weaning were also investigated.

## Materials and methods

### Animal preparation

Sprague-Dawley (SD) rats were bred at the animal unit of Faculty of Medicine, Khon Kaen University and maintained at constant humidity (60 ± 5%), temperature (24 ± 1^o^ C), and light cycle (06.00-18.00 h). Female SD rats were fed normal rat chow with 3% beta-alanine (taurine depletion, TD), 3% taurine (taurine supplementation, TS) or water alone (Control, C) from conception to weaning. Thereafter, the male offspring were fed normal rat chow and tap water *ad libitum*. Further, the male offspring drank either 5% glucose in tap water (TD with glucose, TDG; TS with glucose, TSG; C with glucose, CG) or tap water alone (TDW, TSW, and CW) throughout the experiment. 5% glucose was previously reported to alter renal function before hypertension and diabetes mellitus [25] and to increase the sympathetic nerve activity in TDG rats [24]. All experimental procedures were preapproved by the Khon Kaen University Animal Care and Use Committee and were conducted in accordance with the National Institutes of Health guidelines.

### Experimental protocol

At 7-8 weeks of age, rats were anesthetized with thiopental sodium (50 mg/kg, i.p.). After catheterization of femoral artery for arterial measurement and blood sampling and femoral vein for infusions, arterial pressure was continuously monitored (BIOPAC, Goleta, CA, USA). A tracheal tube was slowly and carefully inserted through the mouth. After a 15-20 min acclimation and while still under deep anesthesia, cardiac arrest was induced by a direct tracheal tube clamping. When the heart arrested and mean arterial pressure went below 60 mm Hg, the clamp was released and cardiopulmonary resuscitation (CPR) was immediately performed by manual chest compression at 60 times/min. Ventilation was continuously controlled by a ventilator through the tracheal tube. The return of arterial pressure pulse and voluntary respiration indicated the success of CPR (within 4 min). Two days later, arterial pressure pulses were continuously recorded in a conscious condition before and during infusion of phenylephrine (increased arterial pressure) or sodium nitroprusside (decreased arterial pressure) to assess the baroreflex sensitivity.

One-day later, male rats were anesthetized with thiopental sodium, tracheostomized, and arterial pulse recorded continuously. Body temperature was servo-controlled at 37±0.5°C by a rectal probe connected to a temperature regulator controlling an overhead heating lamp. Laparotomy was done by longitudinal incision on the mid line area of abdomen. Then, the right renal artery was exposed and a stainless steel electrode (12 MΩ, 0.01 Taper, A-M system, FL, USA) was slowly inserted to the renal nerve, using a gold-plated electrode holder connected to a micromanipulator. A second electrode was attached to the snipped abdominal muscle close to the recording site. Both electrodes were connected to a DAM-80 differential amplifier (WPI, USA) which amplified (1000x) a band pass (500-1000 Hz) signal via a UM100, MP100, and a computer, respectively (BIOPAC). All data were continuously recorded (sampling rate = 5,000 sample/sec). After these experiments, animals were rested for 30 min. Then, arterial blood samples were obtained and sodium (Na), potassium (K), hematocrit (Hct), blood sugar, creatine kinase–MB (CK-MB), troponin T (Trop-T), aspartate aminotransferase (AST), and N-Terminal prohormone Brain Natriuretic Peptide (NT-proBNP) determinations were made. Finally, all animals were sacrificed and kidney and heart weights were measured.

### Data analyses

All data were analyzed by using the Acknowledge Software (BIOPAC). Changes in renal nerve activity and heart rate per changes in arterial pressure following sodium nitroprusside or phenylephrine infusion were used to measure baroreceptor reflex control of renal nerve activity (BSRN) and heart rate (BRHR). The autonomic nervous system control of arterial pressure was estimated by analyzing the power spectrum density of low (LF, 0.3-0.5 Hz; the sympathetic nerve activity dominant) and high (HF, 0.5-4.0 Hz; the parasympathetic nerve activity dominant) frequency components of arterial pulse (Fourier analysis). The absolute values were normalized by the total power (LF+HF). In addition, all blood chemistry parameters were specifically measured by the Khon Kaen University hospital laboratory.

All data were expressed as mean ± SEM. Statistical comparisons among groups were performed by using one-way ANOVA followed by *post hoc* tests. Duncan’s Multi-Range and paired t-tests were used to indicate significant differences among groups and within groups, respectively (Statmost version 3.5, DataMost, USA). The significant criterion was p< 0.05.

## Results

Compared to CW, body, heart, and kidney weights significantly decreased only in TDW groups. Kidney to body but not heart to body weight ratios also were decreased in TDW (Table 1). This decrement was restored to CW levels by high sugar intake. TSG (compared to CG and TSW rats) displayed slightly increased body and heart weights. At the end of experiment, plasma sodium concentration slightly decreased in TSW, but not in any other group (Table 2). Compared to CW, CG, TDW and TDG displayed significantly higher plasma potassium concentrations but TSW and TSG did not differ from CW rats in this measure. Plasma glucose concentration varied within the normal range for non-fasting rats (75-100 mg/dl) in most groups, but compared to the other groups, TDW and TDG displayed lower and higher values, respectively. Compared to other groups, TDG displayed a sharp rise in BUN and a lower hematocrit. Plasma creatinine levels significantly increased in all perinatal taurine depleted and supplemented rats, those of TDW were lowest, but they were partially restored to control levels by glucose supplementation. All plasma cardiac injury markers displayed higher concentrations in TD, compared to the other groups of the same dietary sugar status (Table 3).

**Table 1:** Body (BW), heart (HW), and kidney (KW) weights among groups

Treatment	BW (g)	HW (g)	KW (g)	HW/BW (%)	KW/BW (%)
CW	190 ± 1	0.78 ± 0.01	0.97 ± 0.01	0.41 ± 0.01	0.51 ± 0.01
CG	192 ± 1	0.82 ± 0.02	0.91 ± 0.02	0.43 ± 0.01	0.47 ± 0.01
TDW	169 ± 1*	0.67 ± 0.01*	0.76 ± 0.01*	0.40 ± 0.01	0.45 ± 0.0*
TDG	190 ± 2^£^	0.83 ± 0.01^£^	0.98 ± 0.01^£^	0.44 ± 0.01^£^	0.52 ± 0.01^£^
TSW	191 ± 2	0.82 ± 0.01*	1.05 ± 0.02	0.43 ± 0.00	0.55 ± 0.01
TSG	204 ± 2^#,€^	0.89 ± 0.01^#,€^	0.96 ± 0.10	0.43 ± 0.00	0.47 ± 0.05^€^

Data are mean SEM (n = 10 each groups). *^,#,€,£^ P < 0.05 when compared to CW*, CG^#^,

TSW^€^, and TDW^£^; see text for abbreviations.

**Table 2:** General blood chemistry profiles in anesthetized animals

Treatment	Plasma Na (mEq/L)	Plasma K (mEq/L)	Blood sugar (mg/dl)	BUN (mg/dl)	Plasma Cr (mg/dl)	Hct (%)
CW	137.30±1.25	4.42±0.11	94.30±1.08	15.61±0.79	0.61±0.06	37.80±0.76
CG	135.80±0.74	5.21±0.15*	91.40±0.85*	15.58±0.59	0.47±0.05	37.30±0.47
TDW	138.30±0.70	5.47±0.10*	78.10±1.29*	17.44±0.83	0.37±0.03*	35.90±0.55*
TDG	136.30±0.72	5.60±0.13*^,#^	98.70±0.82*^,#,£^	27.77±2.31^#,£^	0.52±0.06*^,£^	33.90±0.53*^,#^
TSW	132.30±0.75*	4.73±0.08	88.30±0.99*	14.00±0.80	0.46±0.04*	39.30±0.63
TSG	136.00±0.83^€^	4.57±0.10	85.80±0.76*^,#^	16.10±0.73	0.46±0.05*	39.30±0.47^#^

Data are mean SEM (n = 10 each groups). *^,#,€,£^ P < 0.05 when compared to CW*, CG^#^,

TSW^€^, and TDW^£^; see text for abbreviations.

**Table 3:** Blood cardiac injury markers in anesthetized animals

Treatment	AST (u/l)	CK–MB (u/l)	Trop T (ng/ml)	NT-proBNP (pg/ml)
CW	162.10±5.41	275.00±25.53	<0.01	<5
CG	714.00±51.47*	410.50±28.50*	<0.01	<5
TDW	491.50±9.84*	621.10±37.87*	<0.01	<5
TDG	1471.50±110.24*^,#,£^	1084.40±30.12*^,#,£^	0.07±0.04	7.8±0.41
TSW	171.00±4.44^#^	477.20±25.17*	<0.01	<5
TSG	544.60±65.32*^,#,€^	286.00±29.38^#,€^	<0.01	<5

Data are mean SEM (n = 10 each groups). *^,#,€,£^ P < 0.05 when compared to CW*, CG^#^,

TSW^€^, and TDW^£^; see text for abbreviations.

Prior to the cardiac arrest procedure, MAP of all groups varied between 100 to 120 mm Hg (Table 4); TD rats displayed significantly higher arterial pressure than controls, while TS rats displayed significantly lower arterial pressure than controls. Three days after cardiac arrest resuscitation recovery, all groups displayed significantly increased arterial pressure, and no longer displayed any group differences, except for TSG, which was about 5 mm Hg lower than the arterial pressures in all other groups. Among the groups, TDG displayed a significantly decreased post-reperfusion increase in arterial pressure (3.3% compared to 7-20%). Tachycardia responses to the reperfusion were very similar to the MAP changes, including the finding that TDG displayed the least change (1.4% compared to 6-18%). Baroreflex sensitivity of heart rate and renal sympathetic nerve activity were significantly blunted in TDW and TDG when compared to CW or CG (Table 5). High sugar intake exacerbated this disorder. In contrast, a significant increase in baroreflex sensitivity was observed in perinatal taurine supplemented rats, a condition that was attenuated by high sugar intake. It is worth noting that high glucose intake alone slightly and significantly depressed the baroreflex sensitivity in the CG group.

**Table 4:** Mean arterial pressure and heart rate before and after cardiac arrest in anesthetized animals

Treatment	Mean arterial pressure (mm Hg)			Heart rate (bpm)		
		
	Pre-arrest	Recovery	%	Pre-arrest	Recovery	%
CW	110 ± 2	122 ± 1^π^	10.9	395 ± 5	422 ± 1^π^	6.8
CG	112 ± 2	123 ± 1^π^	9.8	411 ± 2*	483 ± 2*^,π^	17.5
TDW	115 ± 2*	123 ± 1^π^	7.0	408 ± 3*	437 ± 4*^,π^	7.1
TDG	120 ± 1*^,#,£^	124 ± 1^π^	3.3	417 ± 1*	423 ± 2^#,£^	1.4
TSW	102 ± 1*^,#^	123 ± 1^π^	20.6	392 ± 3^#^	427 ± 2^,π^	8.9
TSG	103 ± 1*^,#^	117 ± 1*^,€,π^	13.6	409 ± 1*^,€^	434 ± 2*^,#,€,π^	6.1

Data are mean SEM (n = 10 each groups). *^,#,€,£^ P < 0.05 when compared to CW*, CG^#^,

TSW^€^, and TDW^£^; see text for abbreviations, ^π^ P< 0.05 paired Student t-test of same groups; see text for abbreviations.

**Table 5:** Baroreflex sensitivity control of heart rate (BSHR) and renal nerve activity (BSRA) in conscious animals

Treatment	BSHR (bpm/mm Hg)		BSRA (spikes/mm Hg)	
		
	Phenylephrine	Nitroprusside	Phenylephrine	Nitroprusside
CW	2.40±0.02	3.66±0.02	10.56±0.57	13.75±1.05
CG	2.24±0.01*	3.27±0.25*	7.37±0.02*	13.70±0.04
TDW	1.99±0.05*	3.12±0.12*	8.79±0.58*	10.41±0.74*
TDG	1.96±0.02*^,#^	2.94±0.01*	6.35±0.03*^,£^	5.72±0.02*^,#,£^
TSW	2.89±0.04*	3.94±0.08	12.36±0.67*	14.20±0.77
TSG	2.46±0.01^#,€^	3.84±0.01^#^	9.55±0.02^#,€^	12.42±0.02

Data are mean SEM (n = 10 each groups). *^,#,€,£^ P < 0.05 when compared to CW*, CG^#^,

TSW^€^, and TDW^£^; see text for abbreviations.

**Figure 1:**
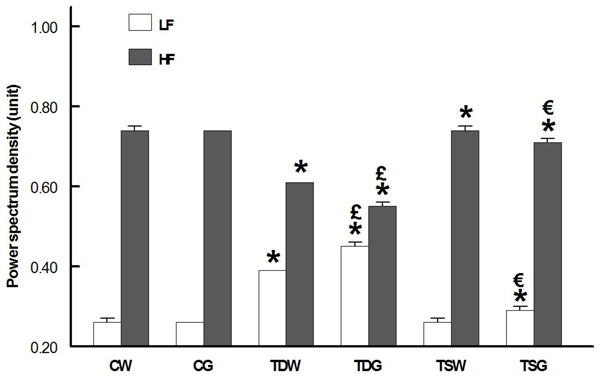
**Low (LF) and high frequency (HF) power spectrum density of arterial pulse in conscious animals** (*P < 0.05 compared to corresponding control (CW or CG), ^£^P < 0.05 compared to TDW, ^€^ P < 0.05 compared to TSW)

Three days after cardiac arrest, sympathetic nerve activity (indirectly estimated by low frequency component of arterial pulse) in CG and TSW was unchanged and was not influenced by glucose. Both glucose and control diet TD rats displayed a significantly elevated low frequency component (Fig. 1). Parasympathetic (high frequency) nerve activity was not altered by the high sugar diet, but it was decreased in the TD groups compared to all other groups. In TS rats, the parasympathetic nerve activity was significantly higher than CW or CG, while the sympathetic nerve activity was remained at CW levels and slightly increased by the high sugar intake.

## Discussion

Previous experiments indicate that perinatal taurine depletion depresses baroreflex sensitivity and the autonomic nervous system activity in adult male rats [24]. A high sugar diet would be expected to exacerbate this baroreflex impairment and increase sympathetic nerve activity. The present data indicate that, three days after cardiac arrest, these abnormal responses are present and markedly amplified. In addition, the present data are the first to demonstrate that perinatal taurine supplementation improves baroreceptor reflex function and maintains near normal autonomic nervous activity. Moreover, perinatal taurine depletion alone increased cardiac injury, a condition that is markedly exacerbated by a high sugar diet.

The decreased (compared to CW) body weight in TDW and the high sugar diet ability to restore body weight in TDG are similar to that previously reported [24]. While the lower body weight in TDW is unlikely to play a major role in post-cardiac arrest changes in this group, since TDG rats had a normal body weight but displayed exacerbated adverse effects of the cardiac arrest/resuscitation. Taurine influences growth and development in the early life. Its perinatal deficiency induces low birth weight and injuries to several body organs [18,19]. The low body weight at birth has been shown to underline a high risk of obesity, diabetes mellitus, hypertension and coronary vascular diseases in the mature life [26-29]. One possible mechanism is due to the body’s propensity to prefer anabolism over catabolism; this is supported by changes in metabolic enzymes in adipose tissues, muscle and liver. This may partially explain why TDG display normal weight as adults. However, the existence of baroreflex sensitivity dysregulation implies that perinatal taurine depletion leads to long-term changes in rats. In contrast, perinatal taurine supplemented rats display changes in baroreflex that are not related to body weight. Thus, low birth weights may not play a key role of cardiovascular system dysregulation following perinatal taurine under- or over-exposure.

Acute cardiac arrest produces hypotension. Resuscitation, while saving the organism, leads to reperfusion, which initiates mechanisms that damage the heart [30-32]. These mechanisms likely include increased calcium entry into cardiac myocytes, sympathetic nerve hyperactivity, superoxide generation, and release of adverse enzymes. Taurine content increases early in the cardiac recovery period and taurine supplementation before or after cardiac ischemia has been shown to protect the ischemia/reperfusion injury [12,16,17]. Baroreflex sensitivity also blunted while arterial pressure rises probably as a result of sympathetic nerve hyperactivity and subsequently increased total peripheral resistance [32,33]. Although all these common phenomena were also observed in the present experiment, some amplification was significantly noted.

We previously reported that perinatal taurine depletion alone depressed autonomic nerve activity in adult male rats and this was reversed and over activated by a high sugar diet [24]. The high sugar treatment alone does not have any effect on the nerve activity or baroreflex sensitivity. In the present study, TD rats displayed sympathetic nerve overactivity, suggesting that these changes are related to ischemia/reperfusion. Blunted baroreflex function was also exacerbated by TD, as supported by a consistent decrease in baroreflex sensitivity control of both heart rate and renal nerve activity tested by either phenylephrine or sodium nitropusside. Non cardiac ischemia/reperfusion TDW depressed mainly that control of heart rate while that control of renal nerve activity significantly decreased only that tested by sodium nitroprusside infusion [24]. In addition, the high sugar intake exaggerated sympathetic nerve hyperactivity in the present study. The cardiovascular responses to ischemia/reperfusion recovery are thus amplified by perinatal taurine depletion. Interestingly, TD decreased parasympathetic nerve activity while it increased sympathetic nerve activity.

All cardiac injury markers increased in TDG animals when compared to CW and other groups, suggesting increased myocardial damage in these animals [34-37]. These largest increases also suggest that a combination of high sugar intake and perinatal taurine depletion has the greatest impact on ischemia/reperfusion-induced myocardial damage. Elevated troponin-T and NT-proBNP confirmed significant ventricular injury in these animals compared to all other groups. These markers are released mainly from the cardiac ventricle [36,37]. The rise in plasma potassium levels in TDG also may indicate that myocardial damage and metabolic acidosis during the early phase of ischemia/reperfusion recovery, are potentiated in these animals [38]. In contrast to perinatal taurine depletion, perinatal taurine supplementation appears to have negligible effect on myocardial injury. It is noted that AST and CK-MB are not specific markers for myocardial injury; liver or muscle damage can also elevate their plasma levels [34,35]. Troponin-T and NT-proBNP are much more specific markers for cardiac damage [39,40].

Three days after cardiac arrest/resuscitation recovery, MAP and HR significantly increased in all rats. These changes are not consistent with blunted baroreflex and heightened sympathetic nerve activity in the TD compared to CG or TSG rats. The rise in arterial pressure in response to ischemia/reperfusion appears to be initiated by cardiac sympathetic afferent nerve from the ischemic or infracted heart tissue [32]. Increased vascular resistance and heart rate determines arterial pressure in this condition. Further, sympathetic nerve hyperactivity is related to the size of the ischemia or infract [30-33]. In the present study, the increased plasma cardiac injury marker levels suggest that TDG animals develop more myocardial damage or larger infarct size than other groups. Thus, this injury likely activates sympathetic nerve hyperactivity more than in other groups.

The high glucose intake significantly increased BUN in TD rats more than other groups. This might be due to tissue damage rather than severe renal glomerular damage since plasma creatinine levels were not different from those of CW or CG groups. In general, the plasma creatinine increases in case of decreased glomerular filtration and/or muscle damage [41]. Non-fasting blood sugar concentrations remained within normal range (70-110 mg/dl) and the minor differences among groups did not correlate with changes in cardiovascular responses.

## Conclusions

Taurine is the main free amino acid in cardiac muscle and plays many protective roles for the heart. The present study indicates that perinatal taurine depletion and high sugar intake exacerbates ischemia/reperfusion induced cardiovascular dysregulation in adult male rats, and the depletion appears to predispose these animals to increased myocardial damage and decreased recovery.

## List of abbreviations used

CW: control with water intake alone; CG: control with high sugar intake; TDW: perinatal taurine depletion (TD) with water intake alone; TDG: perinatal taurine depletion with high sugar intake; TSW: perinatal taurine supplementation (TS) with water intake alone; TSG: perinatal taurine supplementation with high sugar intake; BW: body weight; HW: heart weight; KW: kidney weight; SD: Sprague Dawley; i.p.: intraperitoneal; BSRA: baroreceptor reflex sensitivity control of renal nerve activity; BSHR: baroreceptor reflex sensitivity control of heart rate; SEM: standard error of means; BUN: blood urea nitrogen; Hct: hematocrit; K: potassium: Na: sodium; Cr: creatinine; CK-MB: creatine kinase-MB: Trop-T: troponin-T; CPR: cardiopulmonary resuscitation; AST: aspartate aminotransferase; NT-proBNP: N-Terminal prohormone Brain Natriuretic Peptide; HF: high frequency; LH: low frequency; bpm: beat per minute

## Competing interests

The authors declare that they have no competing interests.

## Authors’ contributions

1. Supaporn Kulthinee: research proposal preparation, data collection and analysis, article preparation

2. J. Michael Wyss: research consult, article preparation

3. Dusit Jirakulsomchok: research consult, article preparation

4. Sanya Roysommuti: research proposal design, data analysis, article preparation, correspondence
